# Diagnostic Model of In-Hospital Mortality in Patients with Acute ST-Segment Elevation Myocardial Infarction Used Artificial Intelligence Methods

**DOI:** 10.1155/2022/8758617

**Published:** 2022-05-25

**Authors:** Yong Li

**Affiliations:** ^1^No.2 Clinic, Beijing Anzhen Hospital, Capital Medical University, Beijing, China; ^2^Datun Community Health Service Center, Chaoyang District, Beijing, China

## Abstract

**Background:**

Preventing in-hospital mortality in patients with ST-segment elevation myocardial infarction (STEMI) is a crucial step.

**Objectives:**

The objective of our research was to develop and externally validate the diagnostic model of in-hospital mortality in acute STEMI patients used artificial intelligence methods.

**Methods:**

We divided nonrandomly the American population with acute STEMI into a training set, a test set, and a validation set. We converted the unbalanced data into balanced data. We used artificial intelligence methods to develop and externally validate several diagnostic models. We used confusion matrix combined with the area under the receiver operating characteristic curve (AUC) to evaluate the pros and cons of the above models.

**Results:**

The strongest predictors of in-hospital mortality were age, gender, cardiogenic shock, atrial fibrillation (AF), ventricular fibrillation (VF), third degree atrioventricular block, in-hospital bleeding, underwent percutaneous coronary intervention (PCI) during hospitalization, underwent coronary artery bypass grafting (CABG) during hospitalization, hypertension history, diabetes history, and myocardial infarction history. The F2 score of logistic regression in the training set, the test set, and the validation dataset was 0.81, 0.6, and 0.59, respectively. The AUC of logistic regression in the training set, the test set, and the validation data set was 0.77, 0.78, and 0.8, respectively. The diagnostic model built by logistic regression was the best.

**Conclusion:**

The strongest predictors of in-hospital mortality were age, gender, cardiogenic shock, AF, VF, third degree atrioventricular block, in-hospital bleeding, underwent PCI during hospitalization, underwent CABG during hospitalization, hypertension history, diabetes history, and myocardial infarction history. We had used artificial intelligence methods developed and externally validated several diagnostic models of in-hospital mortality in acute STEMI patients. The diagnostic model built by logistic regression was the best. We registered this study with the registration number ChiCTR1900027129 (the WHO International Clinical Trials Registry Platform (ICTRP) on 1 November 2019).

## 1. Introduction

In the United States, an estimated 605000 acute myocardial infarction (AMI) events occur each year [1]. In Europe, the in-hospital mortality of patients with ST-segment elevation myocardial infarction (STEMI) is between 4% and 12% [2]. Coronary heart disease including STEMI remains the main cause of death [1]. Preventing in-hospital mortality of STEMI is a crucial step. A tool is needed to help early detection of patients with increased in-hospital mortality. The Global Registration Risk Score for Acute Coronary Events (GRACE) can be accessed via mobile devices, so it enjoyed a high reputation among users. Myocardial infarction thrombolysis (TIMI) risk score can predict the clinical manifestations of 30-day mortality in patients with fibrinolytic-eligible STEMI [3]. The ACTION (acute coronary treatment and intervention outcomes network) score [4] was established in 2011 using 65668 AMI patients, and 16336 AMI patients were used to validate as a model for predicting in-hospital mortality. The ACTION model updated in 2016 used more patients and added cardiac arrest as a risk factor [5]. Xiang Li used the machine learning method to make a prediction model of in-hospital mortality for STEMI patients [6]. Kwon JM used deep learning to establish a prediction model of in-hospital mortality in STEMI patients, which is better than the GRACE score and TIMI score [7].

The current prediction models had the following problems: people had insufficient understanding of the dataset of in-hospital mortality as unbalanced data. The unbalanced data were not converted into balanced data. There was no confusion matrix to be made, and the area under the receiver operating characteristic curve (AUC) or *C* statistic to evaluate the prediction model was not comprehensive. Traditional statistical methods were difficult to deal with the above problems calmly; artificial intelligence methods were needed.

The objective of our research was to develop and externally validate the diagnostic model of in-hospital mortality in acute STEMI patients used artificial intelligence methods.

## 2. Methods

The training dataset was 44996 patients with acute STEMI from January 2016 to December 2016 in the United States. The test dataset was 43581 hospitalized patients with acute STEMI from January 2017 to December 2017 in the United States. The validation dataset came from 40498 hospitalized patients with acute STEMI from January 2018 to December 2018 in the United States. Data from the National (Nationwide) Inpatient Sample (NIS) were used for this study.

Inclusion criteria were as follows: all those STEMI patients who were hospitalized and all those STEMI patients over 18 years of age. Exclusion criteria were none. It was a retrospective analysis, and informed consent was waived by Ethics Committee of Beijing Anzhen Hospital Capital Medical University. Outcome of interest was in-hospital mortality. In-hospital mortality was defined as cardiogenic or noncardiogenic death during hospitalization. The presence or absence of in-hospital mortality was decided, blinded to the predictor variables and based on the medical record. We selected 14 predictor variables according to clinical relevance. Fourteen potential candidate variables were age, gender, cardiogenic shock, atrial fibrillation (AF), ventricular fibrillation (VF), first degree atrioventricular block, second degree atrioventricular block, third degree atrioventricular block, in-hospital bleeding, underwent percutaneous coronary intervention (PCI) during hospitalization, underwent coronary artery bypass grafting (CABG) during hospitalization, hypertension history, diabetes history, and myocardial infarction history. All of them were based on the medical record and blinded to the predictor variables. AF was defined as all types of AF during hospitalization. VF was defined as all types of VF during hospitalization. In-hospital bleeding was defined as all types of bleeding during hospitalization.

We kept all continuous data as continuous and retained on the original scale. We used univariable and multivariable logistic regression models to identify the correlates of in-hospital mortality. We entered all variables of Table 1 into the univariable logistic regression. Based on the variables significantly generated by univariate logistic regression, we constructed a multivariate logistic regression model using the backward variable selection method. We used the Akaike information criterion (AIC) and Bayesian information criterion (BIC) to select predictors. It accounts for model fit while penalizing for the number of parameters being estimated and corresponds to using *α* = 0.157 [8].

In the training dataset, 5169 out of 44966 hospitalized patients (11.5%) experienced in-hospital mortality which represented an imbalanced dataset. We evaluated the effect of common sampling methods including downsampling methods. Therefore, the downsampling technique was additionally implemented on the original dataset to create 1 balanced dataset. We randomly selected 13% in the survival data as the control group. This ultimately yielded 2 datasets: original and downsampling.

To ensure reliability of data, we excluded patient who had missing information on predictors. Discrimination was the ability of the diagnostic model to differentiate between patient with and without in-hospital mortality. This measure was quantiﬁed by calculating the AUC [8] and confusion matrix.

Predictive classifiers were developed based on data from the training set using 5 supervised artificial intelligence methods: logistic regression, random forest, extreme gradient boosting (XGBoost), *K* nearest neighbour classification model, and multilayer perceptron. Confusion matrix includes accuracy, sensitivity, specificity, precision, F1 score, and F2 score. TP, true positive; FN, false negative; FP, false positive; TN, true negative = TN. Accuracy = (TP + TN)/(TP + TN + FP + FN); sensitivity = recall = TP/(TP + FN); specificity = TN/(TN + FP); precision = TP/(TP + FP); F1 score = 2^*∗*^precision^*∗*^recall/(precision + recall), F2 score = 5^*∗*^precision^*∗*^recall/(4^*∗*^precision + recall). F1 score was defined as the harmonic average of precision and recall. In addition to F1 scores, F2 score and F 0.5 score were also widely used in statistics. Among them, in the F2 score, the weight of the recall was higher than the precision, and in the F 0.5 score, the weight of the precision was higher than the recall. The weight of the recall was higher than the precision for the mortality in STEMI patients. We used F2 score combined with AUC to evaluate the pros and cons of the above models.

We performed statistical analyses with STATA version 15.1 (StataCorp, College Station, TX).

We performed artificial intelligence statistical analysis using Python 3.8.5, Pandas 1.2.1, Sklearn 0.0, NumPy 1.19.2, and Keras 2.4.3.

## 3. Results

The patients' baseline characteristics of original and downsampling are given in Table 1. Twelve variables (age, gender, cardiogenic shock, AF, VF, third degree atrioventricular block, in-hospital bleeding, underwent PCI during hospitalization, underwent CABG during hospitalization, hypertension history, diabetes history, and myocardial infarction history) were significant differences in the two groups of patients (*P* < 0.157). After application of the backward variable selection method, AIC and BIC, all of them remained as significant independent predictors of in-hospital mortality. Results are given in Table 2. In the test set, 4895 out of 43581 hospitalized patients (11.2%) experienced in-hospital mortality. The baseline characteristics of the patients are given in Table 1. In the validation dataset, 4001 out of 40498 hospitalized patients (9.9%) experienced in-hospital mortality. The baseline characteristics of the patients are given in Table 1. By comparing F2 score and AUC of Table 3, we can find that the diagnostic model built by the dataset of downsampling was better than those of the diagnostic model built by the dataset of original. By comparing F2 score and AUC of Table 3, we can find that the diagnostic model built by logistic regression was better than the diagnostic model built by decision tree, XGBoost, multilayer perceptron, and *K* nearest neighbour. So, we used the diagnostic model built by dataset of downsampling and built by logistic regression (modellog.m Supplementary Materials).

The code used for using the diagnostic mode can be seen in code 1 Supplementary Materials.

  We input the following code on the browser: https://127.0.0.1:8000/ml/predict? AGE = 60&FEMALE = 1&HBP = 1&VF = 0&AF = 1&OMI = 1&CSHOCK = 1&IIIAVB = 1&DM = 1&PCI = 1&CABG = 0&BLEEDING = 1
  The result can be as follows: ({“features”: {“AF”: 1.0, “AGE”: -1.0, “BLEEDING”: 1.0, “CABG”: 0.0, “CSHOCK”: 1.0, “DM”: 1.0, “FEMALE”: 1.0, “HBP”: 1.0, “IIIAVB”: 1.0, “OMI”: 1.0, “PCI”: 1.0, “VF”: 0.0}, “result”: 0}, {“message”: “1 = death,0 = alive”})

## 4. Discussion

In this study, we investigated the predisposing factors of in-hospital mortality in patients with acute STEMI. Age, gender, cardiogenic shock, AF, VF, third degree atrioventricular block, in-hospital bleeding, underwent PCI during hospitalization, underwent CABG during hospitalization, hypertension history, diabetes history, and myocardial infarction history were the significant independent predictors of in-hospital mortality.

The F2 score of logistic regression in the training set, the test set, and the validation dataset was 0.8, 0.6, and 0.6, respectively. The AUC of logistic regression in the training set, the test set, and the validation dataset was 0.77, 0.78, and 0.8, respectively. The diagnostic model built by logistic regression was the best. So, we use the diagnostic model built by logistic regression.

Granger CB et al. observed that age, Killip class, systolic blood pressure, ST-segment deviation, cardiac arrest during presentation, serum creatinine level, positive initial cardiac enzyme findings, and heart rate were the independent predictors of in-hospital mortality among 11389 patients in the GRACE [9]. Karen S. Pieper et al. generated the updated GRACE risk model and a nomogram [10]. The GRACE risk model has since been upgraded again [11] and simplified [12]. TIMI risk score predicted 30-day mortality at presentation of fibrinolytic-eligible patients with STEMI [3]. C-ACS [13] was simple four-variable score that have been developed to enable risk stratification at first medical contact. ACTION score [4] used 65668 patients to develop and 16336 patients to validate a model to predict in-hospital mortality. The ACTION model updated in 2016 used more patients (243440) and added cardiac arrest as a risk factor [5]. This was a form of internal validation because their cohorts were randomly created [8]. Xiang Li used the machine learning method to make a prediction model of in-hospital mortality for STEMI patients [6]. Kwon JM used deep learning to establish a prediction model of in-hospital mortality in STEMI patients [7].

So far, clinicians and researchers usually use GRACE or TIMI scores to guide treatment decisions. Our diagnostic model of in-hospital mortality was built upon these studies in several ways. We converted the unbalanced data into balanced data. We used the confusion matrix combined with AUC to evaluate the pros and cons of the models.

Our study has several important limitations including its retrospective nature. We used PCI and CABG during hospitalization as one of the baseline variables and predictors of in-hospital mortality. Therefore, there may be selection bias towards survivors and may not be clinically useful as predictor of survival (e.g., for clinical use to identify high-risk patients for more intensive treatment). Information such as number of hospitals, PCI vs. non-PCI centers, location of hospitals (i.e., rural vs. academic centers), and primary vs. rescue PCI were not included in the analysis. Variables such as electrocardiographic parameters, heart rate, presenting blood pressure, Killip class, creatinine, chronic renal failure, acute kidney injury, time to presentation, weight, biomarker levels, and location of culprit lesions were not included in the analysis either. The F2 score and AUC of logistic regression in the training set, the test set, and the validation dataset were the modest.

## 5. Conclusion

The strongest predictors of in-hospital mortality were age, gender, cardiogenic shock, AF, VF, third degree atrioventricular block, in-hospital bleeding, underwent PCI during hospitalization, underwent CABG during hospitalization, hypertension history, diabetes history, and myocardial infarction history. We had used artificial intelligence methods developed and externally validated several diagnostic models of in-hospital mortality in acute STEMI patients. The diagnostic model built by logistic regression was the best.

## Figures and Tables

**Table 1:** Demographic and clinical characteristics of patient with and without in-hospital mortality.

Characteristic	Training datasets								Test datasets				Validation datasets			
	Original				Downsampling											
	Total (*n* = 44966)	In-hospital deaths (*n* = 5169)	In-hospital survivors (*n* = 39827)	*P* value	Total (*n* = 10347)	In-hospital deaths (*n* = 5169)	In-hospital survivors (*n* = 5178)	*P* value	Total (*n* = 43581)	In-hospital deaths (*n* = 4895)	In-hospital survivors (*n* = 38686)	*P* value	Total (*n* = 40498)	In-hospital deaths (*n* = 4001)	In-hospital survivors (*n* = 36497)	*P* value
Age (year, *x* ± *s*) (18, 90)	65 ± 14	72 ± 14	64 ± 13	<0.001	68 ± 14	72 ± 14	64 ± 13	<0.001	64 ± 13	71 ± 13	64 ± 13	<0.001	64 ± 13	71 ± 13	64 ± 13	<0.001
Female sex, *n* (%)	15109 (33.6)	2245 (43.4)	12864 (32.3)	<0.001	3940 (38.1)	2245 (43.4)	1695 (32.7)	<0.001	14211 (32.6)	2011 (41.1)	12200 (31.5)	<0.001	12827 (31.7)	1608 (40.2)	11219 (30.7)	<0.001
Medical history, *n* (%)																
Hypertension	32570 (72.4)	3404 (65.9)	29166 (73.3)	<0.001	7201 (69.6)	3,404 (65.9)	3797 (73.3)	<0.001	30182 (69.3)	2,920 (59.7)	27262 (70.5)	<0.001	28411 (70.2)	2365 (59.1)	26046 (71.3)	<0.001
Old myocardial infarction	5260 (11.7)	530 (10.3)	4730 (11.9)	0.001	1121 (10.8)	530 (10.3)	591 (11.4)	0.058	5088 (11.7)	406 (8.3)	4682 (12.1)	0.001	5048 (12.5)	391 (9.8)	4657 (12.8)	0.001
Diabetes	2479 (5.6)	495 (9.6)	1984 (5)	<0.001	786 (7.6)	495 (9.6)	291 (5.6)	<0.001	3844 (8.9)	751 (15.3)	3093 (8)	<0.001	3804 (9.4)	705 (17.6)	3099 (8.5)	<0.001
Cardiogenic shock, *n*_(%)_	5632 (12.5)	2077 (40.2)	3555 (8.9)	<0.001	2542 (24.6)	2077 (40.2)	465 (9)	<0.001	5658 (13)	2012 (41.1)	3646 (9.4)	<0.001	5742 (14.2)	2,024 (50.6)	3718 (10.2)	<0.001
AF^a^, *n* (%)	3173 (7.1)	414 (8)	2759 (6.9)	0.004	771 (7.5)	414 (8)	357 (6.9)	0.031	3300 (7.6)	436 (8.9)	2864 (7.4)	0.004	3204 (7.9)	379 (9.5)	2825 (7.8)	0.004
VF^b^, *n* (%)	3403 (7.6)	975 (18.9)	2428 (6.1)	<0.001	1266 (12.2)	975 (18.9)	291 (5.6)	<0.001	3431 (7.9)	1010 (20.6)	2421 (6.3)	<0.001	3336 (8.2)	931 (23.3)	2405 (6.6)	<0.001
In-hospital bleeding, *n* (%)	1053 (2.3)	343 (6.6)	710 (1.8)	<0.001	429 (4.1)	343 (6.6)	86 (1.6)	<0.001	822 (1.9)	306 (6.3)	516 (1.3)	<0.001	643 (1.6)	296 (7.4)	347 (1)	<0.001
III AVB^c^, *n* (%)	1136 (2.5)	228 (4.4)	908 (2.3)	<0.001	357 (3.5)	228 (4.4)	129 (2.5)	<0.001	1158 (2.7)	236 (4.8)	922 (2.4)	<0.001	1187 (2.9)	267 (6.7)	920 (2.5)	<0.001
II AVB^c^, *n* (%)	332 (0.7)	31 (0.6)	301 (0.8)	0.219	68 (0.7)	31 (0.6)	37 (0.7)	0.470	321 (0.7)	31 (0.6)	290 (0.7)	0.370	313 (0.8)	29 (0.7)	284 (0.8)	0.715
I AVB^c^, *n* (%)	364 (0.8)	46 (0.9)	318 (0.8)	0.490	87 (0.8)	46 (0.9)	41 (0.8)	0.585	369 (0.8)	40 (0.8)	329 (0.9)	0.811	369 (0.9)	30 (0.7)	339 (0.9)	0.259
PCI^d^, *n* (%)	29110 (64.7)	1611 (31.2)	27499 (69)	<0.001	5158 (49.9)	1611 (31.2)	3547 (68.5)	<0.001	29364 (67.4)	1677 (34.3)	27687 (71.6)	<0.001	29246 (72.2)	1,595 (39.9)	27651 (75.8)	<0.001
CABG^e^, *n* (%)	2397 (5.3)	148 (2.9)	2249 (5.6)	<0.001	442 (4.3)	148 (2.9)	294 (5.7)	<0.001	2244 (5.1)	166 (3.4)	2078 (5.4)	<0.001	1940 (4.8)	129 (3.2)	1811 (5)	<0.001

^a^AF, atrial fibrillation. ^b^VF, ventricular fibrillation. ^c^AVB, atrioventricular block. ^d^PCI, underwent percutaneous coronary intervention during hospitalization. ^e^CABG, underwent coronary artery bypass grafting during hospitalization.

**Table 2:** Predictor of in-hospital mortality obtained from multivariable logistic regression models in the training datasets.

In-hospital mortality	Odds ratio					Coef				
	Odds ratio	Std. error	*z*	*P* > *z*	95% Conf. interval	Coef.	Std. error	*z*	*P* > *z*	95% CI Conf. interval
Age	1.035432	0.0013867	26.00	<0.001	1.032718∼1.038153	0.0348187	0.0013393	26.00	<0.001	0.0321938∼0.0374436
Female sex	1.110608	0.0393301	2.96	0.003	1.036136∼1.190431	0.1049072	0.0354132	2.96	0.003	0.0354987∼0.1743157
Medical history										
Hypertension	0.7176797	0.0262568	−9.07	<0.001	0.668019∼0.7710321	−0.331732	0.0365857	−9.07	<0.001	−0.4034387∼−0.2600253
Old myocardial infarction	0.8129311	0.0448676	−3.75	<0.001	0.7295817∼9058027	−0.2071089	0.0551924	−3.75	<0.001	−0.315284∼−0.0989338
Diabetes	1.383109	0.08354	5.37	<0.001	1.228694∼1.55693	0.3243337	0.0604002	5.37	<0.001	0.2059515∼–0.4427159
Cardiogenic shock	7.635366	0.3090266	50.23	<0.001	7.053085∼8.265718	2.032791	0.0404731	50.23	<0.001	1.953465∼–2.112117
AF^a^	0.6754272	0.0420343	−6.31	<0.001	0.5978678∼0.7630481	−0.3924099	0.0622336	−6.31	<0.001	−0.5143855∼–−0.2704342
VF^b^	3.479356	0.1777195	24.41	<0.001	3.147901∼–3.845713	1.246847	0.0510783	24.41	<0.001	1.146736∼–1.346959
In-hospital bleeding	2.466731	0.1898968	11.73	<0.001	2.121258∼–2.868468	0.9028937	0.0769832	11.73	<0.001	0.7520094∼–1.053778
III AVB^c^	1.263896	0.1131563	2.62	0.009	1.060483∼–1.506327	0.2341991	0.0895297	2.62	0.009	0.058724∼–0.4096741
PCI^d^	0.1650676	0.0063145	−47.09	<0.001	0.1531441∼–0.1779195	−1.8014	0.0382538	−47.09	<0.001	−1.876376∼−1.726424
CABG^e^	0.1784059	0.0171668	−17.91	<0.001	0.1477419∼–0.2154342	−1.723694	0.0962234	−17.91	<0.001	−1.912288∼−1.5351
_cons	0.020919	0.0020729	−39.02	<0.001	0.0172263∼–0.0254033	−3.867096	0.0990936	−39.02	<0.001	−4.061316∼−3.672876

^a^AF, atrial fibrillation. ^b^VF, ventricular fibrillation. ^c^AVB, atrioventricular block. ^d^PCI, underwent percutaneous coronary intervention during hospitalization. ^e^CABG, underwent coronary artery bypass grafting during hospitalization.

**Table 3:** Confusion matrix and AUC.

		Original to original											Downsampling to original										
		TP^a^	TN^b^	FP^c^	FN^d^	Accuracy	Recall	Specificity	Precision	*F*1 score	*F*2 score	AUC	TP^a^	TN^b^	FP^c^	FN^d^	Accuracy	Recall	Specificity	Precision	*F*1 score	*F*2 score	AUC
Logistic regression	Training	1039	39074	753	4130	0.89	0.20	0.98	0.58	0.30	0.23	0.59	4243	3775	1403	926	0.77	0.82	0.73	0.75	0.78	0.81	0.77
	Test	987	37928	758	3908	0.89	0.20	0.98	0.57	0.30	0.23	0.59	3979	28944	9742	916	0.76	0.81	0.75	0.29	0.43	0.60	0.78
	Validation	994	35764	733	3007	0.91	0.25	0.98	0.58	0.35	0.28	0.61	3294	28094	8403	707	0.78	0.82	0.77	0.28	0.42	0.59	0.80

Decision tree	Training	1361	39274	553	3808	0.90	0.26	0.99	0.71	0.38	0.30	0.62	4316	4022	1156	853	0.81	0.83	0.78	0.79	0.81	0.83	0.81
	Test	997	37792	894	3898	0.89	0.20	0.98	0.53	0.29	0.23	0.59	3735	28647	10039	1160	0.74	0.76	0.74	0.27	0.40	0.56	0.75
	Validation	892	35570	927	3109	0.90	0.22	0.97	0.49	0.31	0.25	0.60	3094	27765	8732	907	0.76	0.77	0.76	0.26	0.39	0.56	0.77

Xgboost	Training	1339	39251	576	3830	0.90	0.26	0.99	0.70	0.38	0.30	0.62	4496	3904	1274	673	0.81	0.87	0.75	0.78	0.82	0.85	0.81
	Test	1041	37888	798	3854	0.89	0.21	0.98	0.57	0.31	0.24	0.60	4011	28110	10576	884	0.74	0.82	0.73	0.27	0.41	0.59	0.77
	Validation	892	35570	927	3109	0.90	0.22	0.97	0.49	0.31	0.25	0.60	3306	27253	9244	695	0.75	0.83	0.75	0.26	0.40	0.58	0.79

*K* nearest neighbour	Training	370	39689	138	4799	0.89	0.07	1.00	0.73	0.13	0.09	0.53	3699	4128	1050	1470	0.76	0.72	0.80	0.78	0.75	0.73	0.76
	Test	280	38539	147	4615	0.89	0.06	1.00	0.66	0.11	0.07	0.53	3246	31054	7632	1649	0.79	0.66	0.80	0.30	0.41	0.53	0.73
	Validation	290	36332	165	3711	0.90	0.07	1.00	0.64	0.13	0.09	0.53	2678	29935	6562	1323	0.81	0.67	0.82	0.29	0.40	0.53	0.74

Multilayer perceptron	Training	1082	39062	765	4087	0.89	0.21	0.98	0.59	0.31	0.24	0.60	4398	3634	1544	771	0.78	0.85	0.70	0.74	0.79	0.83	0.78
	Test	1043	37929	757	3852	0.89	0.21	0.98	0.58	0.31	0.24	0.60	4129	28018	10668	766	0.74	0.84	0.72	0.28	0.42	0.60	0.74
	Validation	804	35996	501	3197	0.91	0.20	0.99	0.62	0.30	0.23	0.59	3477	26476	10021	524	0.74	0.87	0.73	0.26	0.40	0.59	0.74

## Data Availability

The data generated or analysed during this study are included within the article (and its supplementary information files).

## Abbreviations

AF: Atrial fibrillation
AMI: Acute myocardial infarction
AUC: Area under the receiver operating characteristic curve
FN: False negative
FP: False positive
MI: Myocardial infarction
NIS: National (Nationwide) Inpatient Sample
ROC: Receiver operating characteristic
STEMI: ST-elevation myocardial infarction
TN: True negative
TP: True positive.

## Supplementary Materials

Supplementary Materials The data are demographic and clinical characteristics of patients with acute STEMI. AGE, age; AF, atrial fibrillation; BLEEDING, in-hospital bleeding; CABG, underwent coronary artery bypass grafting during hospitalization; CSHOCK, cardiogenic shock; DIED, in-hospital mortality; DM, diabetes history; FEMALE, female; HBP, history of hypertension; OMI, history of myocardial infarction; PCI, percutaneous coronary intervention during hospitalization; VF, ventricular fibrillation. Data from the National (Nationwide) Inpatient Sample (NIS) data from January 2016 to December 2018 in the United States were used for this study (https://www.hcup-us.ahrq.gov/).
