# Supplementary material for: Diagnostic Model of In-Hospital Mortality in Patients with Acute ST-Segment Elevation Myocardial Infarction Used Artificial Intelligence Methods
Source: Cardiol Res Pract. 2022 May 25;2022:8758617. doi: 10.1155/2022/8758617 (PMC9159851; doi:10.1155/2022/8758617)
Supplement: Supplementary Materials — The data are demographic and clinical characteristics of patients with acute STEMI. AGE, age; AF, atrial fibrillation; BLEEDING, in-hospital bleeding; CABG, underwent coronary artery bypass grafting during hospitalization; CSHOCK, cardiogenic shock; DIED, in-hospital mortality; DM, diabetes history; FEMALE, female; HBP, history of hypertension; OMI, history of myocardial infarction; PCI, percutaneous coronary intervention during hospitalization; VF, ventricular fibrillation. Data from the National (Nationwide) Inpatient Sample (NIS) data from January 2016 to December 2018 in the United States were used for this study (https://www.hcup-us.ahrq.gov/). [file 8758617.f1.zip › Supplementary Materials/code1.docx]

# import joblib

# import flask

# from flask import Flask, request, url_for, Response

# app = Flask(__name__)

# model2 = joblib.load("modellog.m")

# @app.route("/", methods=["GET"])

# def index():

# with app.test_request_context():

# result = {"predict": {"url": url_for("predict"),

# "params": ["AGE", "FEMALE", "HBP", "VF", "AF", "OMI", "CSHOCK", "IIIAVB","DM","PCI","CABG"," BLEEDING"]}}

# result_body = flask.json.dumps(result)

# return Response(result_body, mimetype="application/json")

# @app.route("/ml/predict", methods=["GET"])

# def predict():

# request_args = request.args

# if not request_args:

# result = {"message": "Please write down the features like as http://127.0.0.1:8000/ml/predict?AGE=80&FEMALE=1&HBP=1&VF=1&AF=1&OMI=1&CSHOCK=0&IIIAVB=0&DM=0&PCI=0&CABG=0&BLEEDING=0"}

# result_body = flask.json.dumps(result, ensure_ascii=False)

# return Response(result_body, mimetype="application/json")

# AGE = float(request_args.get("AGE", "-1"))

# FEMALE = float(request_args.get("FEMALE", "-1"))

# HBP = float(request_args.get("HBP", "-1"))

# VF = float(request_args.get("VF", -1))

# AF = float(request_args.get("AF", -1))

# OMI = float(request_args.get("OMI", -1))

# CSHOCK = float(request_args.get("CSHOCK", -1))

# IIIAVB = float(request_args.get("IIIAVB", -1))

# DM = float(request_args.get("DM", -1))

# PCI = float(request_args.get("PCI", -1))

# CABG = float(request_args.get("CABG", -1))

# BLEEDING = float(request_args.get("BLEEDING", -1))

# vec = [[AGE,FEMALE,HBP, VF,AF,OMI,CSHOCK,IIIAVB,DM,PCI,CABG,BLEEDING]]

# print("vec: {0}".format(vec))

# predict_result = int(model2.predict(vec)[0])

# print("predict_result: {0}".format(predict_result))

# result = {

# "features": {

# "AGE": AGE,

# "FEMALE": FEMALE,

# "HBP": HBP,

# "VF": VF,

# "AF": AF,

# "OMI": OMI,

# "CSHOCK": CSHOCK,

# "IIIAVB": IIIAVB,

# "DM": DM,

# "PCI": PCI,

# "CABG": CABG,

# "BLEEDING": BLEEDING

# },

# "result": predict_result },{"message": "1=death,0=alive"}

# result_body = flask.json.dumps(result, ensure_ascii=False)

# return Response(result_body, mimetype="application/json")

# if __name__ == "__main__":

# app.run(port=8000)
